# Increased Serum Hyaluronic Acid and Heparan Sulfate in Dengue Fever: Association with Plasma Leakage and Disease Severity

**DOI:** 10.1038/srep46191

**Published:** 2017-04-10

**Authors:** Tommy Hing-Cheung Tang, Sylvie Alonso, Lisa Fong-Poh Ng, Tun-Linn Thein, Vincent Jun-Xiong Pang, Yee-Sin Leo, David Chien-Boon Lye, Tsin-Wen Yeo

**Affiliations:** 1 Division of Infectious Diseases, Department of Medicine, Queen Elizabeth Hospital, Hong Kong SAR; 2 Department of Microbiology, Yong Loo Lin School of Medicine and Immunology programme, Life Sciences Institute, National University of Singapore, Singapore; 3 Laboratory of Microbial Immunity, Singapore Immunology Network (SIgN), A*STAR, Singapore; 4 Communicable Disease Centre, Institute of Infectious Diseases and Epidemiology, Tan Tock Seng Hospital, Singapore; 5 Saw Swee Hock School of Public Health, National University of Singapore, Singapore; 6 Yong Loo Lin School of Medicine, National University of Singapore, Singapore; 7 Lee Kong Chian School of Medicine, Nanyang Technological University, Singapore

## Abstract

Plasma leakage is a major pathogenic mechanism of severe dengue, but the etiology remains unclear. The association between endothelial glycocalyx integrity and vascular permeability in older adults with dengue has not been evaluated. A prospective cohort study of adults with undifferentiated fever screened for dengue by RT-PCR or NS1 antigen testing was performed. Patients were assessed daily while symptomatic and at convalescence. Serum hyaluronic acid (HA), heparan sulfate (HS) and selected cytokines (TNF-α, IL-6, IL-10) were measured on enrollment and convalescence. Patients were diagnosed as dengue fever (DF, n = 30), dengue hemorrhagic fever (DHF, n = 20) and non-dengue (ND) febrile illness (n = 11). Acute HA and HS levels were significantly higher in all dengue patients compared to ND (p = 0.0033 and p = 0.0441 respectively), but not different between DF and DHF (p = 0.3426 and p = 0.9180 respectively). Enrolment HA inversely correlated with serum albumin, protein and platelets in all dengue and DHF (p < 0.05). HA and HS in all dengue patients decreased significantly at convalescence. Serum IL-10 was significantly associated with HA in all dengue patients (p = 0.002). Serum HA and HS levels were increased in adult dengue and HA was associated with markers of disease severity. Endothelial glycocalyx damage may have a role in vascular leakage in dengue.

Dengue is the most common and rapidly spreading arthropod borne virus, responsible for an estimated 96 million cases globally in 2013^1^. In the last half-century, it has become endemic in many tropical areas and a major public health problem in South-East Asia^1^,^2^,^3^. Increasing migration, travel and global warming suggests dengue may extend to newer areas in the future, with increased incidence from temperate areas including parts of Europe and the United States of America.

Dengue is a systemic febrile illness caused by 4 serotypes of the dengue virus (DENV-1-4) under the genus *Flavivirus*^1^,^2^. Most DENV infections are mild or asymptomatic; however, a small proportion can develop severe disease. The 1997 and 2009 World Health Organization (WHO) guidelines classified dengue into several subgroups according to severity; those without complications are diagnosed with dengue fever (DF), while those with more severe disease are classified as dengue hemorrhagic fever (DHF) or severe dengue (SD)^4^,^5^. In both the 1997 and 2009 classifications, plasma leakage is a major criteria in the definition of DHF and SD^4^,^5^. Historically, complicated dengue had a fatality rate as high as 20%^6^, which was reduced to less than 1% with current management which focuses on adequate fluid replacement^1^,^2^. However, the low fatality rate should not be underestimated when considering the large global burden of disease^3^.

Currently, the pathogenesis of DHF is still not fully understood. Pathophysiological studies of children with dengue shock 50 years ago and recent reports suggest transient increased microvascular permeability and plasma leakage are major pathogenic mechanisms^6^,^7^. *In vitro* studies of dengue have shown viral replication or increased pro-inflammatory cytokines result in endothelial cell damage with increased vascular permeability^8^,^9^,^10^. Recent studies also suggest the DENV nonstructural protein 1 (NS1) may also play an important role in these pathological changes. In a murine model, NS1 was shown to directly increase inflammatory cytokine production and disrupt endothelial cell integrity via a Toll-like receptor 4 (TLR4) mediated mechanism^11^,^12^. In addition, NS1 from all four dengue serotypes was demonstrated to induce human endothelial cell dysfunction and increase permeability *in vitro*^12^.

The glycocalyx is a carbohydrate-rich layer covering the vascular luminal endothelium consisting of a network of interlinked molecules including proteoglycans, glycoproteins, glycosaminoglycans (GAGs) and plasma proteins^13^,^14^. It functions as a semipermeable barrier regulating flow between plasma and interstitial fluid^13^. Hyaluronic acid (HA) and heparan sulfate (HS) are two major constituents of the glycocalyx and break down of these proteoglycans *in vitro* result in increased plasma flow into the interstitium^13^. Pro-inflammatory cytokines including TNF-α, IL-6 and IL-10 which are elevated in dengue can cause glycocalyx shedding^13^,^14^. An *in vitro* study demonstrated NS1 can also affect glycocalyx integrity by increasing expression of endothelial enzymes such as heparanase^15^. In dengue, pediatric studies from Vietnam and Thailand reported increased urinary excretion of HS, and increased serum levels of HA respectively at the time of diagnosis in DSS^16^,^17^. A study of adolescents from Vietnam found increased HS levels in early dengue but did not compare this to a control group^18^. However none of these studies assessed the levels of glycocalyx constituents in older adults with dengue of varying severity or compared this to non-dengue febrile illness.

To investigate the relationships among endothelial glycocalyx, cytokine levels and disease severity in dengue, we conducted a prospective longitudinal study to measure serum HA, HS, IL-6, IL-10 and TNF-α concentrations in adults diagnosed with dengue and other acute febrile illness. We hypothesized that i) HA and HS levels will be higher in dengue compared to non-dengue patients on enrolment, ii) HA and HS levels will be associated with markers of disease severity including albumin and platelets and iii) HA and HS will be associated with pro-inflammatory cytokine levels.

## Methods

### Ethics Statement

The National Healthcare Group Domain Specific Review Board (DSRB-E-2009/432), Singapore approved this study. Written informed consent was taken from enrolled patients and all data were anonymized. All research activities were performed in accordance to study protocol approved by our institutional review board.

### Participants

Between January 2010 to June 2011, adult patients (≥18 years) with acute undifferentiated fever were enrolled as part of a prospective study at the Infectious Disease Research Clinic, Communicable Disease Center, Tan Tock Seng Hospital (TTSH), Singapore as previously reported^19^. Patients were referred from emergency departments or primary care clinics. Epidemiological and clinical data, vital signs and blood samples were collected on standardized data collection forms at enrollment. Epidemiological data included age, gender and ethnicity, clinical data included pre-existing co-morbidities, date of fever onset, symptoms and signs and vital signs included temperature, pulse rate, erect and supine blood pressure.

Patients were followed up daily during the acute febrile and critical phases (up to 7 days from enrollment) and once at convalesce (21–30 days from enrollment). During follow ups and hospitalization, if any, they were reassessed for clinical symptoms and signs.

### Sample Collection and Testing

Dengue virus was detected by real-time polymerase chain reaction (RT-PCR), a two-stage real-time reverse transcriptase polymerase chain reaction comprising screening using SYBR green followed by a tetraplex probe-based serotype detection assay^20^, or NS1 antigen test by Platelia™ NS1 ELISA (Bio-Rad Laboratories, Marnes-la-Coquette, France)^20^,^21^ on serum samples at patients’ first visit. Dengue serology was tested by Panbio^®^ Dengue IgG Indirect, IgG Capture, and IgM Capture ELISAs (Alere Inc., Waltham, MA, USA). Serum samples for HA and HS were taken at enrollment and at convalescence (21–30 days from enrollment). They were processed on the day of collection and stored in a freezer at −70 degrees centigrade. Serum HA and HS was measured by ELISA (Echelon Biosciences, Salt Lake City, Utah, and TSZ Elisa, Massachusetts, USA respectively), and selected serum cytokines including IL-6, IL-10 and TNF-α were measured by Procarta^®^ multiplex immunoassay (Affymetrix, eBioscience, USA). Hematology and biochemistry tests were performed at the Department of Laboratory Medicine (DLM), TTSH.

### Clinical Definitions

Dengue was confirmed with a positive serum RT-PCR or NS1 antigen test. Classification of final dengue status was according to the WHO 1997 definitions. DHF was defined by a history of fever, platelet count <= 100 × 10^9^/L, hemorrhagic manifestations, and plasma leakage^4^. Plasma leakage was defined as hematocrit elevation by >=20% on serial daily monitoring or follow up, or signs such as pleural effusion, ascites, or hypoproteinemia/hypoalbuminemia. Dengue shock syndrome (DSS) was defined by a pulse rate >100/min and a narrowed pulse pressure <20 mmHg; or DHF with systolic blood pressure <90 mmHg. As previously described^22^, confirmed cases with samples that are negative by IgG indirect or capture assay during the acute phase were classified as primary cases. An acute sample positive by IgG Capture or an early acute (on or before day 5 of illness) sample positive by IgG Indirect defined secondary infection status in confirmed dengue cases.

For analysis, patients were categorized into 3 subgroups: (i) acute undifferentiated febrile illness with negative RT-PCR or NS1 tests for dengue (non-dengue (ND) subgroup), (ii) dengue fever (DF subgroup) and (iii) DHF (DHF subgroup).

### Statistical Analysis

In descriptive analyses, number and percentage were used for categorical variables. Median and interquartile range (IQR) were used for continuous variables. Pearson correlation coefficient and Spearman’s rank correlation coefficient were determined according to distribution of values. For comparison of specific groups, chi-squared test, Wilcoxon rank sum test, and Kruskal-Wallis rank sum test were used appropriately.

Statistical tests were carried out in R version 3.31 (R Foundation for Statistical Computing, Vienna, Austria) and GraphPad Prism version 7.0a (GraphPad Software, La Jolla California USA) with 5% level of significance and two-tailed p values.

## Results

### Clinical, Hematological, Biochemical and Microbiological Results

Sixty-one patients were recruited from January 2010 to June 2011 and the demographic, clinical and laboratory results are detailed in Table 1.

According to the WHO 1997 classification, there were 20 with DHF, 30 with DF and 11 with non-dengue febrile illness and 2 (4%) DHF patients had DSS. DENV RT-PCR or NS1 was positive in all dengue patients with serotype 2 (DEN-2) being the predominant strain. Seventy-five percent of DHF and 60% of DF were secondary infections. There was no significant difference in RT-PCR crossing point (Cp) values between DF and DHF groups (p = 0.348) and between primary infection and secondary infection (p = 0.0734). Among all dengue patients the median duration of fever at enrollment was 5 days (IQR 4–5 days). Twenty-eight patients (45.9%) required hospitalization during their illness with a significantly higher number in the DHF group (p < 0.001). The median hospital stay was 3 days (IQR 2.75–5.00 days), and all were managed according to hospital guidelines for dengue. There was a significant difference in usage of intravenous fluid treatment, with higher proportion (14 (70%) out of 20) in DHF patients (p = 0.022). One DHF patient had history of peptic ulcer as a reported co-morbidity and there were no fatalities during the study.

### Hyaluronic Acid and Dengue Severity

**Figure 1:**
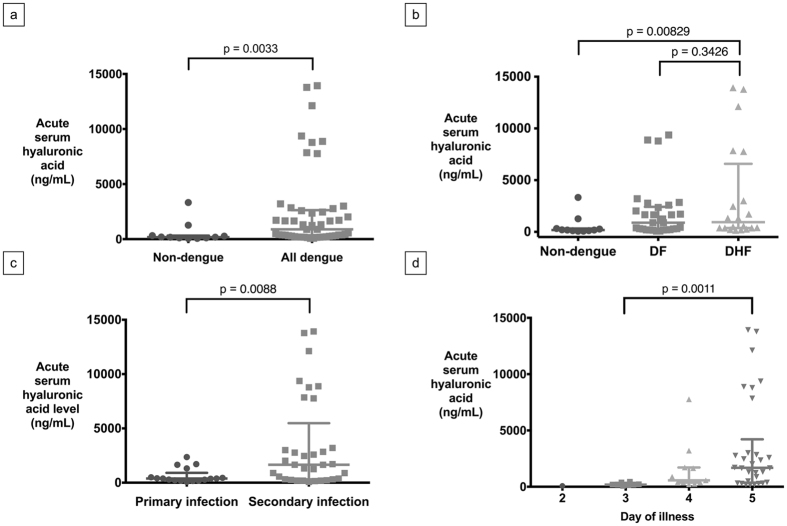
Serum hyaluronic acid (HA) levels in dengue subgroups: (**a**) Acute serum HA in non-dengue and all dengue patients (median, IQR), (**b**) Acute serum HA in non-dengue, DF and DHF subgroups (median, IQR), (**c**) Acute serum HA in patients with dengue primary and secondary infection (median, IQR), (**d**) Acute serum HA with respect to day of illness (first day of illness is defined as the day of fever onset; median, IQR).

**Figure 2:**
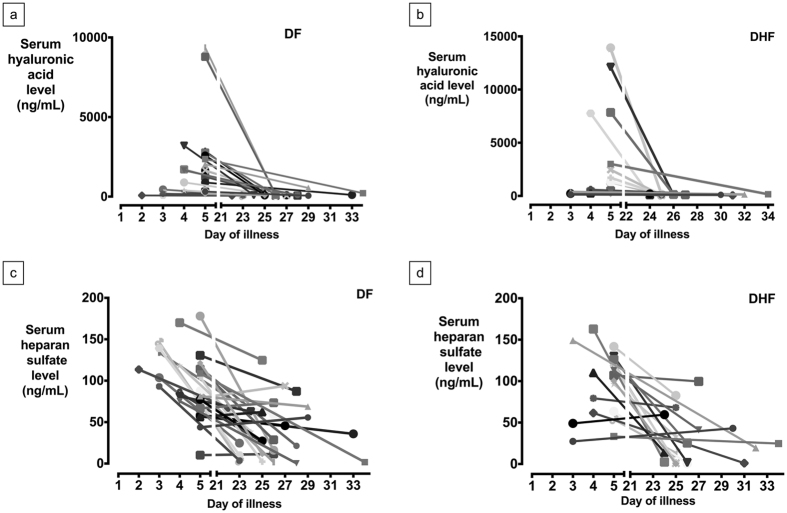
Longitudinal trend of serum hyaluronic acid (HA) and heparan sulfate (HS) level: (**a**) Serum HA in DF group, (**b**) Serum HA in DHF group, (**c**) Serum HS in DF group (1 patient was excluded for outlying values), (**d**) Serum HS in DHF group.

Summary statistics of serum HA and HS are summarized in Table 2. On enrollment, HA levels were significantly higher in all dengue patients (DHF plus DF) compared to non-dengue febrile patients (p = 0.0033) (Fig. 1a). In dengue patients, acute HA levels in DHF patients were not significantly higher than the DF patients (p = 0.3426, Fig. 1b). When analyzed by day of illness, there were also no significant differences between DF and DHF patients enrolled on the third, fourth or fifth day of illness (first day of illness is defined as the day of fever onset). Acute serum HA was significantly higher in patients with secondary DENV infection when compared to those with primary infection (p = 0.0088, Fig. 1c). Patients enrolled on the fifth day of illness had significantly higher HA levels of HA compared to those enrolled on the fourth day, who correspondingly had higher concentrations than those enrolled on the third day, (Fig. 1d). With clinical recovery after discharge, there was a significant decrease in acute serum HA levels compared to convalescent serum HA levels in both DF and DHF groups (p < 0.001, Fig. 2a and b) but not in the non-dengue group (p = 0.6448).

### Heparin Sulfate with Dengue Severity

**Figure 3:**
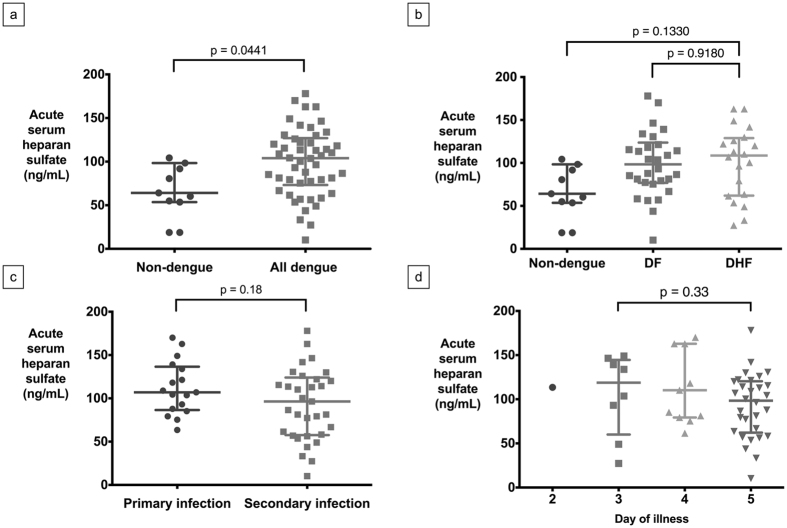
Serum heparan sulfate (HS) levels in dengue subgroups: (**a**) Acute serum HS in all dengue and non-dengue patients (median, IQR; one outlier omitted in each of all dengue and non-dengue groups), (**b**) Acute serum HS in non-dengue, DF and DHF subgroups (median, IQR; one outlier omitted in each of the DF and non-dengue groups), (**c**) Acute serum HS in dengue primary and secondary infection (median, IQR; one outlier omitted in secondary infection), (**d**) Acute serum HS with respect to day of illness (median, IQR; one outlier omitted at the fourth day of illness.

Acute serum HS levels were significantly higher in all dengue patients compared to the ND group p = 0.0441 (Fig. 3a), but there was no significant difference between the DF and DHF patients (p = 0.9180, Fig. 3b). There were also no significant differences between DF and DHF patients enrolled on the third, fourth or fifth day of illness when analyzed by day of illness. No significant difference in HS levels was seen between primary and secondary DENV infections (p = 0.18) (Fig. 3c). There was no significant increase in HS level among the third, fourth and fifth day of illness (p = 0.33, Fig. 3d). However, there was also a significant decrease between acute and convalescent HS levels in both DF and DHF groups (p < 0.001) (Fig. 3c and d), but not in the non-dengue group (p = 0.5564).

### Hyaluronic Acid and Heparan Sulfate with Markers of Disease Severity

In all dengue patients, there were significant negative associations between acute serum HA with serum albumin (Spearman’s rank correlation coefficient (r) = −0.3533, 95% CI −0.5807 to −0.07471, p = 0.0118), total protein (r = −0.3326, 95% CI −0.565 to −0.05138, p = 0.0183) and platelet count (r = −0.5981, 95% CI −0.755 to −0.3764, p < 0.001). In contrast, there was a significant association between HA and DENV PCR Cp (r = 0.33, 95 %CI 0.13 to 0.57, p = 0.02).

Significant inverse association were also found in the DHF subgroup between acute serum HA and acute serum albumin (r = −0.5828, 95% CI −0.8198 to −0.1754, p = 0.0070), globulin (r −0.4964, 95% CI −0.7755 to −0.05509, p = 0.0260), protein (r = −0.6559, 95% CI −0.8551 to −0.2877, p = 0.0017) and platelet count (r = 0.8015, 95% CI −0.9205 to −0.5465, p < 0.0001) (Table 3). The associations in all dengue patients remained significant after adjusting for age, gender and disease severity.

In contrast, the only severe disease parameter significantly correlated with acute serum HS was platelet count in the DHF subgroup (r = −0.4934, 95% CI −0.7739 to −0.05111, p = 0.0270) (Table 4). There were no significant correlations between HA or HS levels with these markers of severity in the non-dengue febrile patients.

### Pro-inflammatory Cytokines

Acute serum IL-6, IL-10 and TNF-α levels were measured in 22 dengue patients (12 DF and 10 DHF patients) either on enrollment (n = 19) or 1 day (n = 3) afterwards. The sampling time was at a median of 5 days after disease onset (IQR 3–6 days). There was no significant difference in IL-6, IL-10 and TNF-α level between DHF and DF subgroups, both during the acute and convalescent phase (Table 2). In dengue patients who had cytokine measurement, acute serum IL-10 level was significantly associated with acute serum HA (r = 0.49, p = 0.012). Among these patients no significant correlation was observed in the DF and DHF groups. There was no significant association between acute HA levels with either IL6 or TNF in all dengue patients with cytokine measurements, or the DF and DHF groups. Acute serum HS was not significantly correlated with IL-6, IL-10 or TNF-α.

## Discussion

The endothelial glycocalyx constituents HA and HS were increased in dengue patients compared to those with non-dengue acute febrile illness. Among dengue patients, HA and HS concentrations were not significantly higher in DHF compared to those with DF. Acute HA but not HS levels were significantly higher in secondary compared to primary DENV infection. In all dengue patients and the DHF subgroup, there was a significant inverse association between HA and, albumin, protein and platelet count. With clinical recovery, there was a significant decrease in the HA and HS levels in DHF and DF patients.

The glycocalyx is a semi-permeable layer overlying the endothelial luminal surface with several homeostatic functions including regulation of solute and plasma movement between the intravascular and interstitial space^13^,^14^. The glycocalyx consists of a network of membrane-bound proteoglycans, glycoproteins and plasma proteins. Damage results in decreased thickness, increased microvascular permeability, decreased mechanotransducer signaling and increased blood cell with vessel wall interaction^13^. Glycocalyx damage is reflected in release of glycosaminoglycan constituents such as HA and HS with increased serum levels^13^,^14^.

Both glycocalyx and albumin are negatively charged, thus the former prevents the latter moving from plasma into the interstitium^13^. About 30–40% of the body’s total albumin is present in the intravascular compartment and accounts for 80% of the colloid oncotic pressure in normal physiological condition^13^. Glycocalyx damage leads to increased movement of albumin across the vascular wall resulting in increased interstitial fluid due to the increased oncotic pressure, and decreased intravascular volume^13^. The elevated HA and HS levels in dengue patients suggest glycocalyx damage might be increased in dengue fever. In this and other dengue studies, decreased albumin and protein due to vascular leakage was associated with worsening disease severity^10^. The significant inverse relationships between HA, with albumin and protein respectively further suggest glycocalyx disruption could increase microvascular permeability and vascular leakage in dengue. Increased HA was also inversely associated with decreased platelet counts, suggesting an additional role in modulating platelet-endothelial cell interactions. The glycocalyx acts as a mechanotransducer to vascular shear stress to induce endothelial nitric oxide release. However, we have found that vascular nitric oxide bioavailability is increased in DHF compared to DF^23^, although other authors have found opposite results^24^. Further studies are required to further define the relationship between the glycocalyx and vascular nitric oxide.

The current results are consistent with studies of adult sepsis, non-dengue viral hemorrhagic fever, and pediatric dengue. In adults with sepsis, circulating glycosaminoglycans including HA, was increased in patients with septic shock and was highest in those with a fatal outcome^25^. Adults with Pumala hantavirus infection had increased syndecan-1 (a marker of endothelial glycocalyx degradation), which was associated with disease severity^26^. In Vietnamese children with DSS, there was a selective increase in the renal clearance of glycosaminoglycans with increased urinary excretion of HS but not chondroitin-4-sulphate or chondroitin-6-sulphate^16^. In Thai children, those with DSS had significantly increased HA levels at the time of diagnosis compared to those with DF and controls^17^. Our results suggest endothelial glycocalyx damage with shedding of glycosaminoglycans may occur prior to the development of DHF. This is supported by a study of Vietnamese adolescents with DF which found increased HS levels during the febrile phase, but this was not compared with DHF, non-dengue or controls^18^. In addition, HA levels were significantly higher in secondary DENV infection while compared to primary infection. This may reflect a stronger inflammatory response known in subsequent DENV infections. The longitudinal decrease in HA levels suggest that glycocalyx repair or recovery parallels clinical recovery.

There was a significant elevation in HS between all dengue patients compared to non-dengue, but no difference between the DF and DHF subgroups, and lack of differential HS increase in secondary DENV infection. While this could reflect the relatively small sample size of the study, there may be other reasons. Adults with septic shock have also been found to have selective increase of several glycosaminoglycans^25^. Another possible explanation is the increased urinary excretion of HS seen in Vietnamese children with dengue may also occur in adults resulting in a lack of measurable differences between DF and DHF. In addition, dengue virus and NS1 protein can bind to HS which is a cell receptor for dengue virus envelope protein^27^,^28^, and the most abundant proteoglycan in endothelium. We postulate that dengue virus could directly interact with the glycocalyx, as suggested by the association between HA and DENV viral load, releasing its constituents like HS and HA into the bloodstream and possibly urine. NS1 binding may also result in a conformational change in HS structure such that it was not measured by the methods used in the study.

The role of pro-inflammatory cytokines in the pathogenesis of DHF or severe dengue has been reported previously with inconsistent results due to different study designs and methods, however, most studies suggests a deleterious role^29^,^30^. In contrast, we did not find significant differences in the levels of TNF-α, IL-6 and IL-10 with increasing dengue severity or plasma leakage. The association between IL-10 and HA in dengue patients suggest the former may increase endothelial glycocalyx damage as suggested by a study in Brazil which found an association between IL-10 and vascular leakage in children with dengue^31^. The lack of increase of both HA and HS in patients with non-dengue acute febrile illness compared to dengue is consistent with a recent report which showed DENV NS1 could compromise the glycocalyx by interacting with endothelial cells^15^.

The main limitation of our study is the small sample size restricting our ability to compare differences with disease severity. Nearly 60% of the patients in the current study presented at day 5 of illness and it is unclear what the HA and HS profile is during the early acute febrile phase of dengue. Increased serum HA is not specific to endothelial glycocalyx damage but may also be seen in joint damage by rheumatological diseases like rheumatoid arthritis. While arthralgia is a common symptom in dengue, frank arthritis with joint damage is rare. Finally, the results may only be applicable to adults as the pathophysiology of severe dengue may differ in pediatric patients. An example is the increased vascular permeability of the blood vessels in children compared to adults.

In summary, in adults with dengue, the glycocalyx components HA and HS were increased compared to non-dengue acute febrile illness. HA was also associated with markers of plasma leakage and disease severity including serum protein, albumin and platelet count. Further studies involving both children and adults with prospective profiling of glycocalyx components are needed to delineate their roles in dengue pathogenesis and potential as prognostic markers. In addition, interventions to attenuate damage or reconstitute the glycocalyx may have therapeutic potential to decrease vascular leakage in dengue^32^.

## Additional Information

**How to cite this article:** Tang, T. H.-C. *et al*. Increased Serum Hyaluronic Acid and Heparan Sulfate in Dengue Fever: Association with Plasma Leakage and Disease Severity. *Sci. Rep.* **7**, 46191; doi: 10.1038/srep46191 (2017).

**Publisher's note:** Springer Nature remains neutral with regard to jurisdictional claims in published maps and institutional affiliations.

## Figures and Tables

**Table 1:** Clinical and biochemical parameters at enrolment.

Categories	DHF (n = 20)	DF (n = 30)	Non-dengue acute febrile illness (n = 11)	p value
Age, years (median, IQR)	42 (34.75–45.00)	33 (28–38)	30 (25–41)	0.010^^^
Male (number, percentage)	16 (80%)	28 (93.33%)	7 (63.64%)	0.065*
Chinese ethnicity (number, percentage)	17 (85%)	25 (83.33%)	7 (63.64%)	0.041*
Co-morbidities (number, percentage)	1 (5%)	0 (0%)	0 (0%)	0.353*
Hospitalization (number, percentage)	16 (80%)	10 (33.33%)	2 (18.18%)	<0.001*
Use of IV fluid (number, percentage)	14 (70%)	9 (30%)	1 (9.09%)	0.022*
Positive DENV RT-PCR (number, percentage)	18 (90%)	27 (90%)	0 (0%)	<0.001*
DENV RT-PCR crossing point (Cp) value	24.56 (20.63–28.4)	25.89 (22.62–29.04)	NA	0.348^#^
Positive dengue NS1 antigen test (number, percentage)	18 (90%)	28 (93.33%)	0 (0%)	<0.001*
Predominant serotype (serotype, number, percentage)	DENV2 (14, 70%)	DENV2 (23, 76.67%)	NA	0.790^#^
Secondary infection rate (number, percentage)	15 (75%)	18 (60%)	NA	0.428^#^
Day of fever on enrolment (median, IQR)	5 (4–5)	5 (4–5)	5 (4–5)	0.98*
Temperature, degrees Celsius on enrolment (median, IQR)	37.60 (37.10–38.00)	37.35 (37.03–38.05)	37 (36.75–37.30)	0.096^^^
Supine SBP, mmHg (median, IQR)	113.5 (110.75–116.25)	116.0 (110.0–120.0)	118.0 (112.5–122.0)	0.550^^^
Supine DBP, mmHg (median, IQR)	72 (62.25–75.25)	69 (65.00–74.75)	71 (65.50–76.50)	0.964^^^
Erect SBP, mmHg (median, IQR)	110 (103.50–116.25)	108.5 (101.0–114.75)	127.0 (106.5–132.5)	0.128^^^
Erect DBP, mmHg (median, IQR)	70 (67.0–78.0)	69 (65.0–75.0)	75 (71.5–80.0)	0.195^^^
Pulse rate (median, IQR)	76.5 (68.0–85.0)	79.5 (70.25–85.5)	76.0 (63.0–77.0)	0.305^^^
Haemoglobin g/dL (median, IQR)	14.9 (14.40–15.50)	15.1 (14.63–16.03)	14.7 (14.35–15.50)	0.318^^^
Haematocrit, percentage (median, IQR)	43.15 (41.45–45.63)	44.15 (42.43–47.03)	42.80 (41.45–45.20)	0.326^^^
Leukocyte x10^9^/L (median, IQR)	2.30 (1.98–2.78)	2.45 (1.88–3.08)	4.10 (3.15–4.70)	<0.001^^^
Neutrophil x10^9^/L (median, IQR)	1.37 (1.12–1.91)	1.22 (0.96–2.03)	2.10 (1.22–2.50)	0.251^^^
Lymphocyte, x10^9^/L (median, IQR)	0.50 (0.45–0.61)	0.60 (0.49–0.79)	1.04 (0.86–1.61)	<0.001^^^
Platelet, x10^9^/L (median, IQR)	71.0 (35.0–92.0)	100.5 (79.0–127.5)	112.0 (100.5–145.0)	<0.001^^^
Creatinine, μmol/L (median, IQR)	72.0 (64.25–79.75)	78.5 (68.25–88.25)	76.0 (60.00–90.00)	0.395^^^
Serum total protein, g/L (median, IQR)	63.0 (60.25–70.25)	70.0 (66.25–72.00)	70.5 (69.00–74.75)	0.016^^^
Serum albumin, g/L (median, IQR)	37.0 (31.75–39.25)	40.0 (38.00–42.00)	39.5 (38.25–40.00)	0.017^^^
Serum globulin, g/L (median, IQR)	26.0 (25.75–31.00)	30.0 (28.00–32.00)	31.5 (30.00–32.75)	0.038^
Serum AST, IU/L (median, IQR)	52 (36.00–81.75)	48 (31.25–74.50)	67 (32.00–101.00)	0.744^^^
Serum ALT, IU/L (median, IQR)	34 (20.50–51.75)	30 (20.25–50.50)	65 (41.00–92.50)	0.048^^^
Serum PT, second (median, IQR)	12.9 (12.15–13.33)	12.9 (12.25–13.65)	13.0 (12.35–13.80)	0.755^^^
Serum aPTT, second (median, IQR)	36.05 (33.15–39.78)	35.40 (33.03–37.83)	31.30 (28.25–32.75)	<0.001^^^

Abbreviations: DHF, dengue hemorrhagic fever (DHF was defined by a history of fever, platelet count <= 100 × 10^9^/L, hemorrhagic manifestations, and plasma leakage; plasma leakage was defined as hematocrit elevation by >=20% on serial daily monitoring or follow up, or signs such as pleural effusion, ascites, or hypoproteinemia/hypoalbuminemia); DF, dengue fever; RT-PCR, reverse transcription polymerase chain reaction; NS1, nonstructural protein 1; DENV-2, dengue serotype 2; NA, not available; IQR, interquartile range; IV, intravenous; SBP, systolic blood pressure; DBP, diastolic blood pressure; AST, aspartate aminotransferase; ALT, alanine transaminase; PT, prothrombin time; aPTT, activated partial thromboplastin time.

Chi-squared test*, Wilcoxon rank sum test^#^, and Kruskal-Wallis rank sum test^^^ were used for calculation of p values.

**Table 2:** Serum hyaluronic acid, heparan sulfate and cytokines.

Categories	DHF (n = 20)	DF (n = 30)	Non-dengue acute febrile illness (n = 11)	p value
Acute serum HA, ng/mL (median, IQR)	935.91 (377.17–4193.12)	886.81 (231.78–2274.15)	117.08 (115.63–296.37)	<0.001
Convalescent serum HA, ng/mL ng/mL (median, IQR)	100.11 (69.11–129.59)	89.89 (66.49–116.88)	100.86 (75.72–123.44)	0.913
Acute serum HS, ng/mL (median, IQR)	108.55 (62.99–126.97)	98.36 (77.80–120.56)	64.14 (54.28–95.07)	0.133
Convalescent serum HS, ng/mL (median, IQR)	16.78 (1.64–41.61)	32.24 (11.51–66.28)	95.07 (70.07–115.13)	<0.001
Acute serum IL-6, pg/mL (binary logarithm) (median, IQR)	2.74 (1.64–3.93)	2.65 (1.78–5.86)	NA*	1
Convalescent IL-6, pg/mL (binary logarithm) (median, IQR)	2.03 (1.35–3.38)	1.57 (0.64–2.88)	NA*	0.470
Acute serum IL-10, pg/mL (binary logarithm) (median, IQR)	5.93 (3.22–8.53)	5.86 (1.69–12.60)	NA*	0.497
Convalescent IL-10, pg/mL (binary logarithm) (median, IQR)	0.71 (0.50–0.75)	0.66 (0.48–0.86)	NA*	0.452
Acute serum TNF-α, pg/mL (binary logarithm) (median, IQR)	2.85 (0.63–6.06)	3.62 (0.10–20.27)	NA*	0.715
Convalescent TNF-α, pg/mL (binary logarithm) (median, IQR)	10.87 (4.44–18.41)	11.19 (1.95–15.49)	NA*	0.795

Abbreviations: DHF, dengue hemorrhagic fever; DF, dengue fever; NA, not available; IQR, interquartile range; HA, hyaluronic acid; HS, heparan sulfate; IL, interleukin; TNF-α, tumor necrosis factor alpha.

*No data in non-dengue patient and healthy control.

**Table 3:** Correlation of acute serum hyaluronic acid and severe disease markers in dengue patients.

	All dengue			DF			DHF		
	r	95% CI	p value	r	95% CI	p value	r	95% CI	p value
Acute serum albumin	−0.3533	−0.5807 to −0.07471	0.0118	−0.1871	−0.521 to 0.1964	0.3222	−0.5828	−0.8198 to −0.1754	0.0070
Acute serum globulin	−0.1322	−0.403 to 0.16	0.3603	0.1424	−0.2402 to 0.4867	0.4530	−0.4964	−0.7755 to −0.05509	0.0260
Acute serum protein	−0.3326	−0.565 to −0.05138	0.0183	−0.04242	−0.406 to 0.3327	0.8239	−0.6559	−0.8551 to −0.2877	0.0017
Acute platelet count	−0.5981	−0.755 to −0.3764	<0.0001	−0.5009	−0.7347 to −0.1607	0.0048	−0.8015	−0.9205 to −0.5465	<0.0001

Abbreviations: r: Spearman’s rank correlation coefficient; CI: confidence intervals.

**Table 4:** Correlation of acute serum heparan sulfate and severe disease markers in dengue patients.

	All dengue			DF			DHF		
	r	95% CI	p value	r	95% CI	p value	r	95% CI	p value
Acute serum albumin	−0.1888	−0.4506 to 0.1029	0.1891	−0.1421	−0.4865 to 0.2404	0.4537	−0.2824	−0.6526 to 0.1965	0.2276
Acute serum globulin	−0.08274	−0.3603 to 0.2083	0.5678	0.08313	−0.2959 to 0.4395	0.6623	−0.2953	−0.6605 to 0.183	0.2062
Acute serum protein	−0.2027	−0.462 to 0.08857	0.1580	−0.08215	−0.4388 to 0.2968	0.6661	−0.3458	−0.6911 to 0.1281	0.1353
Acute platelet count	−0.09667	−0.3725 to 0.1948	0.5042	0.1964	−0.1871 to 0.528	0.2982	−0.4934	−0.7739 to −0.05111	0.0270

Abbreviations: r: Spearman’s rank correlation coefficient; CI: confidence intervals.

## References

[b1] Guzman M. G. & Harris E. Dengue. The Lancet 385, 453–465 (2015). 10.1016/S0140-6736(14)60572-9 25230594

[b2] Simmons C. P., Farrar J. J., van Vinh Chau N. & Wills B. Dengue. N. Engl. J. Med. 366, 1423–1432 (2012). 22494122 10.1056/NEJMra1110265

[b3] Bhatt S. . The global distribution and burden of dengue. Nature 496, 504–507 (2013). 23563266 10.1038/nature12060 PMC3651993

[b4] WHO. Dengue haemorrhagic fever: diagnosis, treatment, prevention and control. 2nd edition. Geneva : World Health Organization. *WHO* (1997). Available at: http://www.who.int/csr/resources/publications/dengue/Denguepublication/en/. (Accessed: 11th August 2015).

[b5] WHO. Dengue: guidelines for diagnosis, treatment, prevention and control. *WHO* (2009). Available at: http://www.who.int/csr/resources/publications/dengue_9789241547871/en/. (Accessed: 11th August 2015). 23762963

[b6] Cohen S. N. & Halstead S. B. Shock associated with dengue infection. I. Clinical and physiologic manifestations of dengue hemorrhagic fever in Thailand, 1964. J. Pediatr. 68, 448–456 (1966). 5903315 10.1016/s0022-3476(66)80249-4

[b7] Bethell D. B. . Noninvasive measurement of microvascular leakage in patients with dengue hemorrhagic fever. Clin. Infect. Dis. 32, 243–253 (2001). 11170914 10.1086/318453

[b8] Dalrymple N. A. & Mackow E. R. Roles for endothelial cells in dengue virus infection. Adv. Virol. 2012, 840654 (2012). 22952474 10.1155/2012/840654 PMC3431041

[b9] Srikiatkhachorn A. & Kelley J. F. Endothelial cells in dengue hemorrhagic fever. Antiviral Res. 109, 160–170 (2014). 25025934 10.1016/j.antiviral.2014.07.005 PMC4148486

[b10] Huy N. T. . Factors associated with dengue shock syndrome: a systematic review and meta-analysis. PLoS Negl. Trop. Dis. 7, e2412 (2013). 24086778 10.1371/journal.pntd.0002412 PMC3784477

[b11] Modhiran N. . Dengue virus NS1 protein activates cells via Toll-like receptor 4 and disrupts endothelial cell monolayer integrity. Sci. Transl. Med. 7, 304ra142-304ra142 (2015). 10.1126/scitranslmed.aaa3863 26355031

[b12] Beatty P. R. . Dengue virus NS1 triggers endothelial permeability and vascular leak that is prevented by NS1 vaccination. Sci. Transl. Med. 7, 304ra141-304ra141 (2015). 10.1126/scitranslmed.aaa3787 26355030

[b13] Reitsma S., Slaaf D. W., Vink H., van Zandvoort M. A. M. J. & oude Egbrink M. G. A. The endothelial glycocalyx: composition, functions, and visualization. Pflugers Arch. 454, 345–359 (2007). 17256154 10.1007/s00424-007-0212-8 PMC1915585

[b14] Becker B. F., Jacob M., Leipert S., Salmon A. H. J. & Chappell D. Degradation of the endothelial glycocalyx in clinical settings: searching for the sheddases. Br. J. Clin. Pharmacol., doi: 10.1111/bcp.12629 (2015). PMC4574825 25778676

[b15] Puerta-Guardo H., Glasner D. R. & Harris E. Dengue Virus NS1 Disrupts the Endothelial Glycocalyx, Leading to Hyperpermeability. PLOS Pathog. 12, e1005738 (2016). 27416066 10.1371/journal.ppat.1005738 PMC4944995

[b16] Wills B. A. . Size and charge characteristics of the protein leak in dengue shock syndrome. J. Infect. Dis. 190, 810–818 (2004). 15272410 10.1086/422754

[b17] Honsawek S. . Increased levels of serum hyaluronan in patients with dengue infection. J. Infect. 54, 225–229 (2007). 16876870 10.1016/j.jinf.2006.06.002

[b18] Tam D. T. H. . Effects of short-course oral corticosteroid therapy in early dengue infection in Vietnamese patients: a randomized, placebo-controlled trial. Clin. Infect. Dis. 55, 1216–1224 (2012). 22865871 10.1093/cid/cis655 PMC3466094

[b19] Vasanwala F. F. . Predictive Value of Proteinuria in Adult Dengue Severity. PLoS Negl Trop Dis 8, e2712 (2014). 24587464 10.1371/journal.pntd.0002712 PMC3930505

[b20] Lai Y.-L. . Cost-effective real-time reverse transcriptase PCR (RT-PCR) to screen for Dengue virus followed by rapid single-tube multiplex RT-PCR for serotyping of the virus. J. Clin. Microbiol. 45, 935–941 (2007). 17215345 10.1128/JCM.01258-06 PMC1829098

[b21] Pok K.-Y., Lai Y.-L., Sng J. & Ng L.-C. Evaluation of nonstructural 1 antigen assays for the diagnosis and surveillance of dengue in Singapore. *Vector Borne Zoonotic Dis*. Larchmt. N 10, 1009–1016 (2010). 10.1089/vbz.2008.0176 PMC2992696 20426686

[b22] Gan V. C. . Diagnosing Dengue at the Point-of-Care: Utility of a Rapid Combined Diagnostic Kit in Singapore. PLoS ONE 9, e90037 (2014). 24646519 10.1371/journal.pone.0090037 PMC3960091

[b23] Thein T.-L. . Association Between Increased Vascular Nitric Oxide Bioavailability and Progression to Dengue Hemorrhagic Fever in Adults. J. Infect. Dis. 212, 711–714 (2015). 25732810 10.1093/infdis/jiv122

[b24] Yacoub S., Wertheim H., Simmons C. P., Screaton G. & Wills B. Microvascular and endothelial function for risk prediction in dengue: an observational study. Lancet Lond. Engl. 385 Suppl 1, S102 (2015). 10.1016/S0140-6736(15)60417-2 26312832

[b25] Nelson A., Berkestedt I. & Bodelsson M. Circulating glycosaminoglycan species in septic shock. Acta Anaesthesiol. Scand. 58, 36–43 (2014). 24341693 10.1111/aas.12223

[b26] Connolly-Andersen A.-M., Thunberg T. & Ahlm C. Endothelial activation and repair during hantavirus infection: association with disease outcome. Open Forum Infect. Dis. 1, ofu027 (2014). 10.1093/ofid/ofu027 PMC4324194 25734100

[b27] Chen Y. . Dengue virus infectivity depends on envelope protein binding to target cell heparan sulfate. Nat. Med. 3, 866–871 (1997). 9256277 10.1038/nm0897-866

[b28] Avirutnan P. . Secreted NS1 of dengue virus attaches to the surface of cells via interactions with heparan sulfate and chondroitin sulfate E. PLoS Pathog. 3, e183 (2007). 18052531 10.1371/journal.ppat.0030183 PMC2092380

[b29] Green S. . Elevated plasma interleukin-10 levels in acute dengue correlate with disease severity. J. Med. Virol. 59, 329–334 (1999). 10502265

[b30] Rothman A. L. Immunity to dengue virus: a tale of original antigenic sin and tropical cytokine storms. Nat. Rev. Immunol. 11, 532–543 (2011). 21760609 10.1038/nri3014

[b31] Ferreira R. A. X. . Circulating cytokines and chemokines associated with plasma leakage and hepatic dysfunction in Brazilian children with dengue fever. Acta Trop. 149, 138–147 (2015). 25944351 10.1016/j.actatropica.2015.04.023

[b32] Ushiyama A., Kataoka H. & Iijima T. Glycocalyx and its involvement in clinical pathophysiologies. J. Intensive Care 4, 59 (2016). 27617097 10.1186/s40560-016-0182-z PMC5017018

