# Author Correction: Increased Serum Hyaluronic Acid and Heparan Sulfate in Dengue Fever: Association with Plasma Leakage and Disease Severity

**DOI:** 10.1038/s41598-021-92487-3

**Published:** 2021-06-21

**Authors:** Tommy Hing-Cheung Tang, Sylvie Alonso, Lisa Fong-Poh Ng, Tun-Linn Thein, Junxiong Pang, Yee-Sin Leo, David Chien-Boon Lye, Tsin-Wen Yeo

**Affiliations:** 1 Division of Infectious Diseases, Department of Medicine, Queen Elizabeth Hospital, Yau Ma Tei, Hong Kong SAR Hong Kong; 2 Department of Microbiology, Yong Loo Lin School of Medicine and Immunology Programme, Life Sciences Institute, National University of Singapore, Singapore, Singapore; 3 Laboratory of Microbial Immunity, Singapore Immunology Network (SIgN), A*STAR, Singapore, Singapore; 4 Communicable Disease Centre, Institute of Infectious Diseases and Epidemiology, Tan Tock Seng Hospital, Singapore, Singapore; 5 Saw Swee Hock School of Public Health, National University of Singapore, Singapore, Singapore; 6 Yong Loo Lin School of Medicine, National University of Singapore, Singapore, Singapore; 7 Lee Kong Chian School of Medicine, Nanyang Technological University, Singapore, Singapore

Correction to: *Scientific Reports* 10.1038/srep46191, published online 10 April 2017

This article contains an error in the spelling of the author Junxiong Pang, which is incorrectly given as Vincent Jun-Xiong Pang.

